# Examining the spatial networks of an urban agglomeration through the lens of multi-dimensional element flow: Evidence from western China

**DOI:** 10.1016/j.heliyon.2023.e20949

**Published:** 2023-10-13

**Authors:** Xiaohui Zhang, Jifei Zhang

**Affiliations:** a Institute of Mountain Hazards and Environment, Chinese Academy of Sciences, Chengdu, 610299, China; b University of Chinese Academy of Sciences, Beijing, 100049, China

**Keywords:** Flow space, Traffic flow, Information flow, Migration flow, Spatial structure, Social network analysis, Chengdu–chongqing urban agglomeration

## Abstract

The Chengdu–Chongqing urban agglomeration (CCUA), as the only national-level city cluster in southwestern China, serves as a strategic support for the Yangtze River Economic Belt and an important demonstration area for promoting new urbanization in the country. The study of the networked characteristics of the CCUA contributes to a systematic understanding of its spatial connectivity patterns, optimization of spatial structure and layout, and promotion of high-quality regional development. In this study, we constructed models for traffic flow, information flow, migration flow, and composite flow to calculate the strength of connections between cities and the total flow of various elements in the CCUA. ArcGIS spatial visualization tools were used to depict the spatial connectivity patterns of the element flows within the CCUA. Additionally, social network analysis methods, including network density, centrality, and cohesive subgroups analysis, were employed to reveal the spatial network structure characteristics of the CCUA. The findings are as follows: (1) The overall structure of the cities within the CCUA is relatively loose, with significant differences in connectivity strength. It exhibits a west-strong and east-weak pattern, with Chengdu-Chongqing, Chengdu-Deyang, Chengdu-Mianyang, and Chengdu-Meishan occupying the top tier, while Zigong and Ya'an have relatively weak connections with other cities. Chengdu and Chongqing have prominent positions in the CCUA, with Chengdu having a more prominent core position compared to Chongqing, resulting in an overall hierarchical distribution of “1 + 1+7 + 7”. (2) The network density of the element flows in the CCUA is relatively low, indicating a generally weak element connectivity. The centrality of cities other than Chengdu and Chongqing is at a moderate to lower level, suggesting a weak overall resource connectivity capacity in the CCUA. (3) Most cities tend to form cohesive subgroups based on geographic proximity, while the cohesive subgroup in Chongqing is still in its early stages of development due to administrative boundaries. The research results quantitatively depict the spatial network structure characteristics of the CCUA, providing theoretical references for its development.

## 1 Introduction

Urban agglomerations are complex networks of cities that emerge in the later stages of urban development. They consist of core cities and some affiliated cities and have a profound impact on urban planning, economic geography, and regional economics [1]. The Chengdu–Chongqing urban agglomeration (CCUA) is the only national urban agglomeration in Southwest China and plays a crucial role in the Yangtze River economic belt. It serve as an important demonstration area for the country's new urbanization efforts [2]. Understanding the network spatial structure of the CCUA is essential for comprehending its spatial connections and guiding the development of urban agglomerations.

As urbanization progress, the flow of elements between cities intensifies, resulting in increasingly intricate and interconnected city network [3]. Previous studies focused on urban agglomerations, economic belts, provinces and counties as key areas for investigating urban spatial structures [[4], [5], [6], [7]]. Researchers have primarily employed the gravity model and social network analysis method [[8], [9], [10]]. However, most studies have taken unidimensional approach, focusing on specific flows such as economic flow [11,12], population migration flow [13,14], traffic flow [[15], [16], [17]], and information flow [[18], [19], [20], [21]]. Few studies have examined the multi-dimensional element flow in urban spatial network structures, and there is no consensus in academia regarding the relative importance of each element flow in composite flow models. In this study, we employ a combined subjective and objective method to assign weights to each element flow when constructing the composite flow model.

Existing studies on the CCUA have analyzed it using single or multi-dimensional element flows. For example, Zhao et al. [22] and Wang et al. [23] examined the network connection modes in the Chengdu–Chongqing region from the perspective of information flow. Sun and Hou [24] investigated the network structure characteristics of the CCUA based on population migration data collected during the Spring Festival. Yao et al. [25]. described the overall spatial pattern of CCUA using economic flow data. Yao et al. [26]. analyzed the spatial structure of Chengdu–Chongqing through the traffic network. Tu et al. [27] explored the spatial network pattern of the CCUA based on economic flow and traffic flow. Economic flow is a crucial indicator for measuring urban economic development. However, previous studies have relied on statistical data, which fails to capture the directional expression of the economy, thus inadequately representing the network structure between cities. Population migration flow reflects the movement of people between cities and influences the spatial structure of urban areas. Traffic flow significantly affects the spatial network structure and expansion of cities as it serves as the conduit for element flows between them. Information flow indicates the level of attention exchanged between cities and greatly influences the pace of urban development.

The coordinated development among cities within urban agglomerations or provincial regions remains an unresolved issue. Currently, there are three main knowledge gaps: 1) Most studies on urban network spatial structures focus on single flow elements and do not utilize composite flow analysis; 2) Existing research predominantly relies on statistical yearbook data to analyze the economic connectivity patterns within urban agglomerations, neglecting the use of flow spatial data; 3) There is limited research that simultaneously considers traffic flow and migration flow to study the spatial structure characteristics of urban agglomerations, which encompasses both population mobility and traffic volume. To address these gaps, this paper conducts a literature review to analyze existing research achievements on urban spatial structures, the progress and frontier issues of studying urban spatial structures from the perspective of element flows were identified. Furthermore, the research perspectives, methods, tools, and approaches used by previous researchers were understood, serving valuable references and insights for further research, expanding ideas and methods.

In this paper, CCUA is selected as the research area, building upon existing studies (Table 1). Three flow elements, namely traffic flow, information flow, and migration flow, are chosen as representatives of the most crucial flows between cities. These elements are used to analyze the spatial structure characteristics of the CCUA from a multi-dimensional perspective. The paper consists of five main parts. The first part provides and introduction to the research background. The second part reviews the development process of the CCUA. The third part described the data and methods used, including an overview of the study area, data sources, and the employed models and methods. The fourth part presents the research results. It begins by analyzing the connectivity strength of the various flow elements, using ArcGIS spatial visualization tools to illustrate the spatial connectivity patterns of the element flows within the CCUA. Network density and centrality are then examined to reveal the spatial structure characteristics of the CCUA. Finally, cohesive subgroup analysis is conducted to explore clustering phenomena in the urban network.

**Table 1:** Main indicators of urban spatial network structure research.

Method/Model	Element flow	Data	Study area	Literature sources
modified gravity model social network analysis	economic flow	statistical yearbook data, ordinary train and high-speed railway data, shortest distance between cities	Shandong Peninsula Urban Agglomeration	Scientia Geographica Sinica (2018)
	economic flow, population flow, information flow traffic flow	statistical yearbook data, Tencent population migration data, Baidu search index, rain frequency and passenger capacity	Wuhan Metropolitan Area	Economic Geography (2021)
social network analysis	migration flow	Tencent population migration data	Cities of China	Journal of Geographical Sciences (2021)
	high-speed rail flow	high-speed rail passenger frequency data	Counties of China	Progress in Geography (2021)
	population flow, logistics, capital flow, information flow	passenger frequency of high-speed rail、bullet train、ordinary train and intercity bus, Logistics company outlets, City bank outlets, Baidu search index	Pearl River Delta Urban Agglomeration	Geographical Research (2019)
gravity model social network analysis	economic flow, information flow	statistical yearbook data, Sample survey data, Census data, The shortest mileage between cities, Baidu search index	Huaihai Economic Zone	Economic Geography (2019)
social network analysis	traffic flow	expressway traffic flow data	Jiangsu Province	Sustainability (2017)
	information flow	Baidu search index	China	ISPRS International Journal of Geo-Information (2021)
	information flow	Baidu search index	Yangtze River Delta	International Journal of Environmental Research and Public Health (2021)

The fifth part concludes the paper and offers discussions. Analyzing the spatial network structure of the CCUA from a flow perspective holds significant important for deepening our understanding of the urban connectivity pattern in the CCUA. It contributes to optimizing the spatial structure and layout of the Chengdu-Chongqing Economic Circle, promoting high-quality and coordinated regional development, and servers as reference for studying the urban network structure of other urban agglomerations.

## 2 The development course of the CCUA

World-class urban agglomerations thrive due to the radiation of core cities and the establishment of a “multi-polar support” system for coordinated development among cities of different sizes [28]. Prominent global examples include the North-East Atlantic Coast Urban Agglomeration, the Five Great Lakes Urban Agglomeration in North America, the Pacific Coast Urban Agglomeration in Japan, the British Urban Agglomeration, the Northwest European Urban Agglomeration, and the Yangtze River Delta Urban Agglomeration. Typically, world-class urban agglomerations undergo a gradual development process that can be divided into three stages: initial isolated development of a individual cities, subsequent development along key axes, and ultimately the formation of a networked urban agglomeration structure [29]. In the case of the London urban agglomeration, for example, significant milestones include the formulation of the “Greater London Plan” in the 1940s, which introduced the concept of “four concentric circles” to guide development. The “New City Act” of 1946 facilitated the construction of eight satellite cities around the London Center. In the 1960s, the “Greater London Development Plan” focused on constructing three major express traffic lines, establishing corridors, and creating three “anti-magnetic attraction centers”. In the 21st century, the London administration developed four editions of the “Greater London Regional Space Development Strategic Plan” (2004, 2008, 2011, and 2016) to address crucial economic, social, transportation, and ecological challenges [30]. These efforts resulted in the evolution of London's urban spatial structure from a single-centre model to a multi-center structure, culminating a networked urban agglomeration structure.

**Fig. 1:**
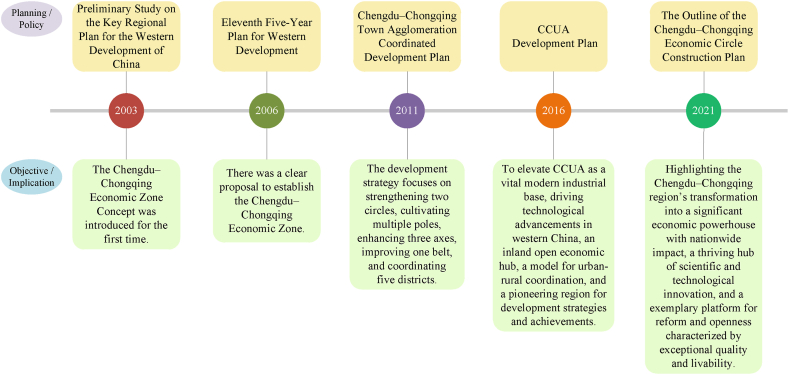
The development history of the Chengdu–Chongqing region.

Chengdu and Chongqing, as the core cities of the CCUA, have faced challenges due to their predominant competition rather than cooperation, resulting in a development setback. Their capacity to attract resources outweighs their ability to distribute them, leading to an imbalanced pattern known as “dual core dominance” and a subsequent “central collapse” within the CCUA [31]. However, a shift in national strategic positioning from the “Chengdu–Chongqing Economic Zone” to the “CCUA” and further to the “Chengdu–Chongqing Dual-City Economic Circle” has highlighted (Table 2 and Fig. 1) the Chengdu–Chongqing region as a world-class economic circle of strategic importance. This transformation aims to drive economic transformation, foster the development of the western region, and actively engage in global competition and cooperation.

**Table 2:** Relevant plans and policies for the Chengdu–Chongqing region.

Time	Planning/Policy	Objective/Implication
2003	Preliminary Study on the Key Regional Plan for the Western Development of China	The Chengdu–Chongqing Economic Zone concept was introduced for the first time.
2004	Research on the Key Economic Belt for the Western Development of China	The Chengdu–Chongqing Economic Zone served as the regional center of the upper Yangtze River economic belt.
2006.12	Eleventh Five-Year Plan for Western Development	There was a clear proposal to establish the Chengdu–Chongqing Economic Zone.
2007.4	Agreement on Promoting the Sichuan-Chongqing Cooperation to Build the Chengdu–Chongqing Economic Zone	The scope of the economic zone was officially defined, emphasizing that Chongqing and Chengdu should jointly lead the efforts to develop the Chengdu–Chongqing Economic Zone into a new growth pole of China.
2010.12	Main Function Zone Plan in China	Functional orientation: National demonstration area for coordinated urban and rural development; Nationally significant high-tech industry hub; Advanced manufacturing and modern service industry base; Science and technology education, business logistics, financial center, and comprehensive transportation hub; Southwest science and technology innovation base; Significant population and economically dense area in the western region.
2011.1	Chengdu–Chongqing Town Agglomeration Coordinated Development Plan	The development strategy focuses on strengthening two circles, cultivating multi-poles, enhancing three axes, improving one belt, and coordinating five districts.
2011.4	Regional Plan for the Chengdu–Chongqing Economic Zone	To transform the region into a significant economic center in west China, a prominent modern industrial base within the country, a pilot area for deepening the inland opening-up initiatives, a demonstration area for coordinating urban and rural development, and a guaranteed area of ecological security in the upper reaches of the Yangtze River.
2014.3	National New Urbanization Plan (2014–2020)	Emphasizing on cultivating Chengdu–Chongqing region and other urban agglomerations, recognizing them as crucial growth poles for promoting balanced land and spatial development and leading regional economic growth.
2015.5	Memorandum on Strengthening the Cooperation Between the Two Provinces and Cities to Build the CCUA	Facilitating the “integration” of transportation, information, and market within CCUA.
2016.4	CCUA Development Plan	To elevate CCUA as a vital modern industrial base, driving technological advancements in western China, an inland open economic hub, a model for urban-rural coordination, and a pioneering region for development strategies and achievements in China.
2018.6	The Action Plan for Deepening Sichuan-Chongqing Cooperation to Promote the Development of the Yangtze River Economic Belt and 12 special cooperation agreements	The CCUA has transitioned from being a conceptual idea to becoming a tangible reality.
2019.4	Key Tasks for New Urbanization Construction in 2019	Acknowledging that the development of the CCUA was implemented concurrently with other major prominent urban agglomerations in China, such as the Beijing-Tianjin-Hebei, Yangtze River Delta, and Guangdong-HongKong-Macao urban agglomeration.
2019.7	Deepening Sichuan-Chongqing Cooperation to Promote the Key Work Plan of the Integrated Development of CCUA	The joint promotion of the integrated development of the CCUA is positioned as a national strategy aiming at building a core engine for the opening and development of the western China.
2020.1	The sixth meeting of the Central Financial and Economic Commission	The concept of a dual-city economic circle in the Chengdu–Chongqing region marked a pivotal moment in its development strategy, envisioning ''two centers and two places '' for growth.
2021.2	The Fourteenth Five-Year Plan for National Economic and Social Development of Sichuan Province and the Outline of Long-term Goals for 2035	Presenting a comprehensive blueprint for the construction and development of the Chengdu–Chongqing economic circle in Sichuan and Chongqing over the next five years.
	The Fourteenth Five-Year Plan for National Economic and Social Development of Chongqing and th Outline of Long-term Goals for 2035	
2021.10	The Outline of the Chengdu–Chongqing Economic Circle Construction Plan	Highlighting the Chengdu–Chongqing region's transformation into a significant economic powerhouse with nationwide impact, a thriving hub of scientific and technological innovation, and a exemplary platform for reform and openness characterized by exceptional quality and livability.
2021.11	Leveraging the key Role of Districts (Cities) and Counties in Reform and Innovation to Foster the Development of the Chengdu–Chongqing Eeconomic Circle (2021–2025)	Refining the tasks and measures: implementing sequential division of responsibilities in the construction of the Chengdu-Chongqing Economic Circle.
2021.11	Chongqing Metropolitan Area Development Plan	To facilitate the integration and promote the growth of both central and western Chongqing, with a strong emphasis on ensuring their harmonious development.
	Chengdu Metropolitan Area Development Plan	Chengdu Metropolitan Area, as the third metropolitan area plan approved at the national level, holds a unique position as the sole plan in central and western China. With a visionary outlook, this plan is committed to establishing a modern metropolis that embodies a thriving economy, a pristine ecological environment, and an exceptional quality of life for its residents.

## 3 Data and method

### 3.1 Study area

**Fig. 2:**
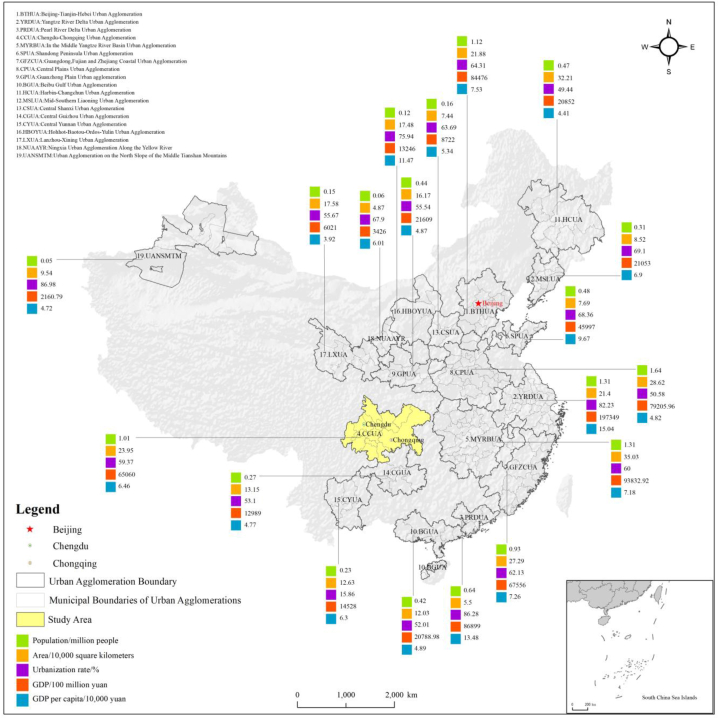
Distribution map of urban agglomerations in China.

The comprises 15 cities in Sichuan and 27 districts and counties in Chongqing. Chengdu and Chongqing, as the core cities, share similarities in population, economy, and transportation facilities (Fig. 2). However, treating the 15 cities in Sichuan and 27 districts and counties in Chongqing as the same level yields to significantly varied results. The average GDP, population, and land area of the 15 cities in Sichuan are 276.36 billion yuan, 4.63 million people, and 10475.76 square kilometers, respectively. In contrast, the average figures for the 27 districts and counties in Chongqing are 77.46 billion yuan, 0.93 million people, and 1593.59 square kilometers, respectively. Due to data collection challenges, Chongqing was considered as a unified research unit for analysis. The research area encompasses 16 cities including Chengdu, Zigong, Luzhou, Deyang, Mianyang, Suining, Neijiang, Leshan, Nanchong, Meishan, Yibin, Guang'an, Dazhou, Ya'an, and Ziyang. Located in southwestern China, the CCUA spans 185,000 square kilometers, representing 1.92 % of China's land area. In 2019, its resident population reached 100.71 million, accounting for 7.19 % of China's population (excluding Hong Kong, Macao, and Taiwan). Additionally, the regional GDP for 2019 amounted to 65,060.45 billion yuan, contributing to 6.57 % of the country’GDP (excluding Hong Kong, Macao, and Taiwan).

### 3.2 Data source

The data used were sourced from various platforms. The baidu index data analysis platform (http://index.baidu.com) provided the information used to represent the information connection between the two cities, using search index data up to August 21, 2019. Data on bus frequency were obtained from the bus network (http://checi.cn/), while ordinary train and high-speed rail passenger frequency data were sourced from the Shengming Timetable. Population migration data were acquired from the Tencent location big data platform (https://heat.qq.com/). It is important to note that the Tencent platform only publishes the top 10 move-in and move-out records for each city, but additional data can be supplemented using move-out and move-in records from other cities beyongd the top 10. For this analysis, data on residents' migration between the 16 cities within the CCUA were collected from January 1, 2019, to August 21, 2019.

### 3.3 Research methods

#### 3.3.1 Traffic flow model

The strength of traffic connections between cities is evaluated based on the passenger frequency of buses, ordinary trains, and high-speed rail. To account for the varying passenger capacities of these three modes of transportation, different scores were assigned to each [32]. Buses and ordinary trains were give a wight of 1, while high-speed rail was assigned a weight of 3. The following formula was used for the caculation:

(1) $${M_{ij}}^{\prime }=\frac{M_{ij}+M_{ji}}{2}$$

(2) $${K_{ij}}^{\prime }=\frac{K_{ij}+K_{ji}}{2}$$

(3) $${G_{ij}}^{\prime }=\frac{G_{ij}+G_{ji}}{2}$$

(4) $$T_{ij}={M_{ij}}^{\prime }+{K_{ij}}^{\prime }+3{G_{ij}}^{\prime }$$

(5) $$T_{i}=\sum_{j=1}^{n}T_{ij}$$

where *T*_*i*_ is the total traffic flow connection in city *i*. *T*_*ij*_ is the strength of the traffic connection between city *i* and city *j*; *M*_*ij*_ ', *K*_*ij*_ ', and *G*_*ij*_*’* are the total bus, ordinary train and high-speed rail traffic connections between city *i* and city *j*; *M*_*i*j_, *K*_*ij*,_ and *G*_*ij*_ are the number of bus, ordinary train, and high-speed rail connections between city *i* and city *j*; and *M*_*ji*_, *K*_*ji*_, and *G*_*ji*_ are the number of bus, ordinary train and high-speed rail connections between city *j* and city *i*.

#### 3.3.2 Information flow model

The Baidu search index quantifies the strength of the information connection between two cities by considering the search volume of internet users. This approach enables the analysis of the information connection pattern between cities. The calculation formula used for this purpose is as follows:

(6) $$C_{ij}=R_{ij}\times R_{ji}$$

(7) $$B_{i}=\sum_{j=1}^{n}C_{ij}$$

where *C*_*ij*_ is the information connection strength between city *i* and city *j*; *B*_*i*_ is the total information flow connection of city *i*; *R*_*ij*_ is the search volume for connections from city i to city *j*; and *R*_*ji*_ is the search volume for connections from city *j* to city *i*.

#### 3.3.3 Migration flow model

The strength of inter-city migration flow is measured by the volume of population migration between cities.

(8) $$L_{ij}=S_{ij}+S_{ji}$$

(9) $$L_{i}=\sum_{j=1}^{n}L_{ij}$$

where *L*_*ij*_ is the migration connection strength between city *i* and city *j*; *S*_*i*j_ is the migration volume from city *i* to city *j*; *S*_*ji*_ is the migration volume from city *j* to city *i*; and *L*_*i*_ is the total number of migration flow connections in city *i*.

#### 3.3.4 Composite flow model

This paper employs the game theory combination weighting method [33,34], incorporating both subjective and objective weightings, to enhance the analysis of traffic flow, information flow and population migration flow. The subjective weighting is achieved through the Analytic Hierarchy Process (AHP), while the objective weighting is accomplished using the CRITIC method.

(1) AHP. It is a subjective weighting analysis method utilized to address the challenges of determining the relative importance of multiple objectives. It quantifies the weights by assessing the relative importance of each objective and employs these weight values to effectively compare their importance. A hierarchical structure model is established, judgment matrices are constructed, and hierarchical single ranking and consistency tests are conducted to ultimately determine the weight [35].
(2) CRITIC method. It is an objective weighting method employed when multiple targets face challenges in effectively measuring the relative importance due to data differences. This method derives the importance of target data bythoroughly exploring and utilizing its own attributes, assigning corresponding weight values to compare the order of importance among various targets. The final weight are determined through dimensionless processing of indicators, calculation of index variability, index conflict, and information amount [36].
(3) Game theory empowerment. The weights obtained through different weighting methods may exhibit significant differences or even conflicts. Therefore, a consistency test of weights is conducted prior to the weighting of game theory combinations. The distance function used for this purpose is as follows:

(10) $$d\left[w_{j\_a}w_{j\_c}\right]={\left\{\frac{1}{2}\sum_{j=1}^{m}{\left[w_{j\_a}-w_{j\_c}\right]}^{2}\right\}}^{\frac{1}{2}}$$

In the formula, $w_{j\_a}$ and $w_{j\_c}$ are the weights of the two groups involved in the game. A smaller value of $d\left[w_{j\_a}w_{j\_c}\right]$ indicates a closer proximity between the weights of the two groups. When $d\left[w_{j\_a}w_{j\_c}\right]$ ＜0.2, it is considered to have passed the consistency test and the weights can be combined.

To minimize the deviation between the combined weight w and the two types of weights $\left(w_{j\_a}{,w}_{j\_c}\right)$, $a_{1}$ and $a_{2}$ are derived basing on the differential property of matrix, and then ${a_{1}}^{*}$ and ${a_{2}}^{*}$ are obtained by normalization. A basic weight vector set $w={\left(w_{j\_a},w_{j\_c}\right)}^{T}$ is established by the AHP and CRITIC method. The linear combination of the vector set is performed as $w=\left(a_{1}{w_{j\_a}}^{T}+a_{2}{w_{j\_c}}^{T}\right)$. The formula is as follows:

(11) $$\left(\begin{array}{cc} w_{j\_a}{w_{j\_a}}^{T} & w_{j\_a}{w_{j\_c}}^{T}\\ w_{j\_c}{w_{j\_a}}^{T} & w_{j\_c}{w_{j\_c}}^{T} \end{array}\right)\left(\begin{array}{c} a_{1}\\ a_{2} \end{array}\right)=\left(\begin{array}{c} w_{j\_a}{w_{j\_a}}^{T}\\ w_{j\_c}{w_{j\_c}}^{T} \end{array}\right)$$

(12) $${a_{1}}^{*}=\frac{a_{1}}{a_{1}+a_{2}}$$

(13) $${a_{2}}^{*}=\frac{a_{2}}{a_{1}+a_{2}}$$

(14) $$\text{Finally}\;\text{calculate}\;\text{the}\;\text{combination}\;\text{weight}\;w=\left({a_{1}}^{*}{w_{j\_a}}^{T}+{a_{2}}^{*}{w_{j\_c}}^{T}\right)$$

The formula for calculating the connection strength and total amount of composite flow is as follows:

(15) $$Q_{ij}=0.34{T_{ij}}^{\prime }+0.22{C_{ij}}^{\prime }+0.44{L_{ij}}^{\prime }$$

(16) $$Q_{i}=\sum_{j=1}Q_{ij}$$

where *Q*_*ij*_ is the composite connection strength of city *i* and city *j*; *Q*_*i*_ is the total amount of composite flow of city *i*; and *T*_*ij’*_, *C*_*ij’*_, and *L*_*ij’*_ represent the result of normalized connection strength of traffic, information, and migration flow between city *i* and city *j*.

#### 3.3.5 Social network analysis (SNA)

Social network analysis is widely utilized to study urban spatial network structures. In the article “Vital Nodes Identification in Complex Networks” [37], the identification of vital nodes in real networks and their roles in structure and function were explored. The article provides a comprehensive review of concepts, metrics, and methods, comparing well-known techniques across networks. Emphasizing physics-based approaches and interdisciplinary solutions, it addressed challenges and future directions, covering both individual vital nodes and sets of vital nodes. Our study examines the spatial network characteristics of the CCUA employing Ucinet, focusing on network density, centrality, and cohesive subgroups. Network density is quantitatively calculated to assess connections between cities. Centrality analysis measures the absorption, radiation, and intermediary capacity of each city using point degree centrality, betweenness centrality, and closeness centrality. The CONCOR Algorithm in Ucinet is used to analyze the number, composition, and distribution of cohesive subgroups within the CCUA.

It is important to note that traditional centrality indices were chosen in this study due to their effectiveness in addressing our specific research question. Centrality indices are widely acknowledged as valuable tools for assessing the significance of nodes in various flows and the dissemination of influence throughout a network. By employing these indices, we can gain insights into the relative importance and interconnectedness of nodes in urban networks, thereby illuminating the spatial structure and functional distribution of cities. While network analysis continues to evolve, the selection of traditional centrality indices aligns well with the objectives of this study.

## 4 Spatial network structure of the CCUA

### 4.1 Connection strength

**Fig. 3:**
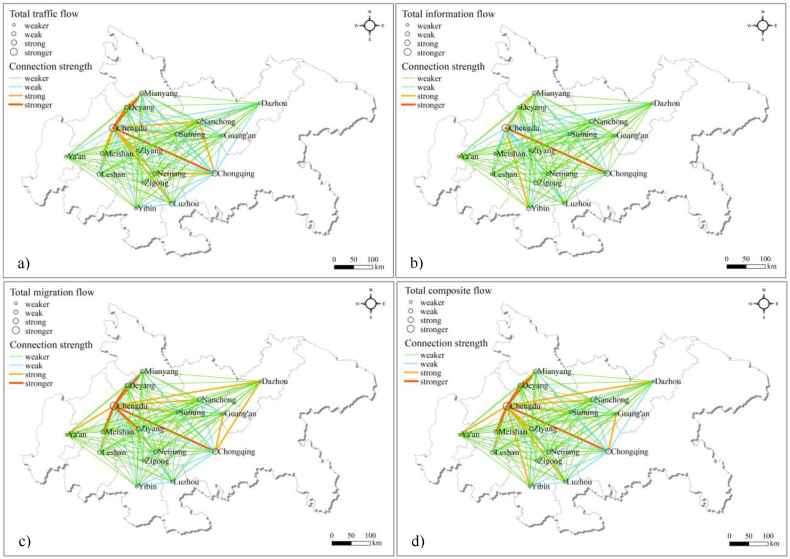
Spatial connection pattern for the CCUA (a) Total traffic flow and its connection strength; b) Total information flow and its connection strength; c) Total migration flow and its connection strength; d) Total composite flow and its connection strength).

The ArcGIS spatial analysis function was utilized to visualize the connection strengths between cities in the CCUA. Employing the natural breakpoint classification method, the connection strengths of the cities in the CCUA were categorized into four levels. The results show that the spatial network structure of the CCUA revolves around Chengdu and Chongqing as the core cities, with notable variations in connection strengths among the cities, indicating significant spatial differentiation (Fig. 3).

#### 4.1.1 Traffic flow

The traffic flow is determined using (1), (2), (3), (4), (5). The cities in the CCUA are categorized into four levels based on total traffic flow: Level 1 includes Chengdu, known for its highly developed traffic system. Chongqing, ranking second in traffic flow and displaying strong connections with other cities, is Level 2. Level 3 encompasses Mianyang, Deyang, Nanchong, Neijiang, Suining, Meishan, Leshan, and Luzhou. Level 4 consists of Ziyang, Dazhou, Yibin, Guang'an, Zigong, and Ya'an. Chengdu and Chongqing jointly account for 41.1 % of the total connections among all cities, reflecting their dominant positions. In terms of traffic connection strength, the stronger connections include Chengdu–Chongqing, Chengdu–Mianyang, and Chengdu–Deyang. Additionally, the strong connections comprise Chengdu–Meishan, Deyang–Mianyang, Chengdu–Luzhou, Chengdu–Suining, Chengdu–Neijiang, Neijiang–Chongqing, Chengdu– Leshan, Chengdu–Nanchong, Nanchong–Chongqing, Chengdu–Ziyang, and Suining–Nanchong. Stronger connections represent 23.3 % of the total traffic connection strength, while strong connections account for 34.5 %. Therefore, the combined strength of stronger and strong connections constitutes 57.8 % of the total strength. Most of these connections are primarily concentrated around Chengdu and Chongqing, indicating weaker connections between other cities. Ongoing efforts aim to establish a 1-h transportation circle connecting the core cities with adjacent and surrounding cities. Chengdu and Chongqing serves as the core cities and transportation hubs within the CCUA. This initiative will stimulate economic development, regional integration, and overall enhancement of the CCUA's comprehensive strength.

#### 4.1.2 Information flow

The calculation of information flow is carried out employing (6), (7). The total information flow in the CCUA is divided into four levels: 1) Chengdu; 2) Chongqing; 3) Mianyang, Yibin, Nanchong, Zigong; and 4) Dazhou, Luzhou, Deyang, Leshan, Suining, Meishan, Neijiang, Guang'an, Ziyang, Ya'an. Chengdu takes the lead, exhibiting the highest network attention and strongest information connection strength with other cities. Chongqing follows closely behind Chengdu in terms of the total information flow and also has demonstrate a high degree of network attention. The combined information flow through Chengdu and Chongqing represents 49.3 % of the total information connection among all cities. Regarding the information connection strength in the CCUA, stronger connections include Chengdu–Chongqing while strong connections comprise Chengdu–Mianyang and Chengdu–Yibin. Stronger connections contributes to 22.5 % of the total strength of information connections between cities, and strong connections account for 13.9 %. Thus, stronger and strong connections constitute a total of 36.4 %. The information flow between Chengdu and Chongqing displays the highest connection strength and holds significant importance within the CCUA. The information flow connection strength between Chengdu and Chongqing exhibits distinct dual-core and multi-node characteristics. Chengdu and Chongqing hold prominent positions and functions, distinguishing them significantly from surrounding cities.

#### 4.1.3 Migration flow

The migration flow within the CCUA is quantified using (8), (9). The four levels of migration flow in the CCUA are: 1) Chengdu; 2) Chongqing; 3) Deyang, Meishan, Ziyang, Mianyang, Nanchong, Neijiang, Leshan; and 4) Suining, Dazhou, Guang'an, Yibin, Luzhou, Zigong, Ya'an. Chengdu exhibits the highest total migration flow and frequent population movement, followed by Chongqing. Together, Chengdu and Chongqing account for 43.2 % of the total connections between cities in the CCUA. Stronger migration connections include Chengdu–Deyang, Chengdu–Meishan, Chengdu–Chongqing, Chengdu–Ziyang, and Chengdu–Mianyang. Strong connections comprise Chengdu–Leshan, Chengdu–Nanchong, Guang'an–Chongqing, Chengdu–Suining, Chengdu–Neijiang, Chengdu–Ya'an, Dazhou–Chongqing, Chengdu – Dazhou. Chengdu–Deyang holds the highest migration strength, followed by Chengdu–Meishan, Chengdu–Chongqing, Chengdu–Ziyang, and Chengdu–Mianyang. Stronger connections contribute to 22.5 % of the total migration strength, while strong connections account for 26.7 %. Overall, stronger and strong connections represent 49.2 % of the total.

#### 4.1.4 Composite flow

Using the game theory combination weighting method as prescribed by (10), (11), (12), (13), (14), the composite flow within the CCUA is determined through (15), (16). The four levels of composite flow in the UCAA are: 1) Chengdu; 2) Chongqing; 3) Mianyang, Deyang, Nanchong, Meishan, Neijiang, Ziyang, Leshan; 4) Suining, Luzhou, Yibin, Dazhou, Guang'an, Zigong, Ya'an. Chengdu exhibits the highest total composite flow and the closest connections with other cities. Chongqing follows Chengdu closely in terms of total composite flow, with stronger connections to other cities as well. The combined composite flow of Chengdu and Chongqing accounts for 43.1 % of all connections. In terms of composite flow connection strength, stronger connections include Chengdu–Chongqing, Chengdu–Deyang, Chengdu–Mianyang, Chengdu–Meishan. Strong connections include: Chengdu–Ziyang, Chengdu–Nanchong, Chengdu–Leshan, Chengdu–Suining, Chengdu–Neijiang, Guang'an–Chongqing, Chengdu–Ya'an, Deyang–Mianyang, Chengdu–Dazhou, Chengdu–Luzhou, Chengdu–Yibin. Stronger connections account for 29.1 % of the total composite connection strength, strong connections account for 32.7 %, and combined stronger and strong connections represent 61.9 % in total. Among the top 30 city pairs, only Deyang–Mianyang, Leshan–Meishan, Suining–Nanchong, Luzhou–Yibin, Zigong–Yibin, Nanchong–Guang'an are not directly connected to Chengdu and Chongqing. Overall, the composite flow network in the CCUA aligns with the single element flow network, especially the traffic flow. Each city in the CCUA exhibits varying levels of connections with other cities. Connections in the western region are stronger compared to the eastern region. Chengdu and Chongqing play significant radiation and driving roles, with looser overall urban spatial connections. Inter-city connections in the CCUA are still influenced by administrative boundaries and spatial distances. Apart from strong connections with Chengdu, Chongqing has not established strong cross-regional connections with other cities in the CCUA.

### 4.2 Urban network density and centrality

#### 4.2.1 Network density

The network densities of traffic flow, information flow, migration flow, and composite flow are 0.25, 0.262, 0.246, and 0.258, respectively. Theses relatively low densities suggest that the flow space network within the CCUA is in its early stage of development, and the connections between cities are relatively loose.

**Fig. 4:**
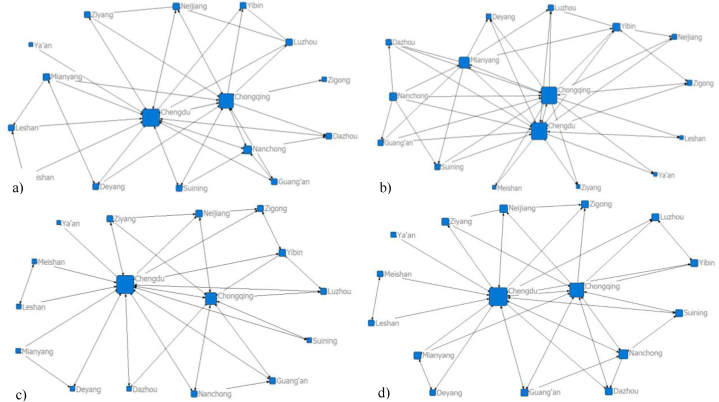
Spatial network structure of the CCUA (a) The network of traffic flow; b) The network of information flow; c) The network of migration flow; d) The network of composite flow).

Cities play a crucial role as nodes in the formation and development of the urban network. They determines the direction and strength of element flows and influence the overall development and trends of the city network [38]. In Fig. 4, Chengdu exhibits the highest connectivity, with the largest radiation range and influence. This reflects Chengdu's extensive impact on transportation, information, and population migration within the CCUA. Chongqing follows closely with significant connectivity and influence. On the other hand, Ya'an shows the poorest connectivity, having limited connections with other cities and a low level of mutual engagement. It indicates lower levels of urban economy, transportation, and overall urban development in Ya'an. Those observations highlight the significant differences between core cities and smaller or medium-sized cities, leading to polarization. The overall structure of the CCUA displays loose connectivitye, weak element connections, and limited closeness among nodes in the urban spatial network. Therefore, it is necessary to leverage the radiation effects of Chongqing and Chengdu as core cities to nurture and expand regional central cities and drive the development of surrounding smaller cities.

#### 4.2.2 Point degree centrality

Centrality is a measure of an individual's influence within a network. In directed networks, a high in-degree indicates strong agglomeration ability, rendering the actor more “important”. Conversely, a high out-degree signifies greater radiation capability, making the actor more “influential” [39].

The point-in degree measures node agglomeration ability. Notably, as depicted in Table 3, Chengdu and Chongqing exhibit high point-in degrees, indicating that the transportation, information, and population migration in these cities are significantly influenced by other cities. They benefit greatly from the interconnections between urban transportation, information, and population migration. Conversely, Deyang, Leshan, Meishan and Ya'an display low point-in degrees, indicating that these cities are influenced by fewer cities in terms of traffic, information, and population migration. Consequently, their benefits from urban interconnections are limited.

**Table 3:** CCUA point degree centrality.

City	Traffic flow		Information flow		Migration flow		Composite flow	
	point-in degree	point-out degree	point-in degree	point-out degree	point-in degree	point-out degree	point-in degree	point-out degree
Chengdu	15	13	13	15	15	15	15	15
Zigong	1	1	3	3	3	3	3	2
Luzhou	1	4	4	1	3	3	3	3
Deyang	3	3	3	1	2	2	2	2
Mianyang	3	4	3	9	2	2	3	3
Suining	3	3	4	1	2	2	3	3
Neijiang	3	3	3	1	4	4	3	4
Leshan	3	2	2	1	2	2	2	2
Nanchong	5	4	3	6	3	3	4	5
Meishan	2	2	2	1	2	2	2	2
Yibin	3	1	4	5	3	4	3	2
Guang'an	2	3	4	1	3	3	3	3
Dazhou	3	2	4	2	2	2	3	2
Ya'an	1	1	2	0	1	1	1	1
Ziyang	3	2	2	1	3	3	3	2
Chongqing	9	12	7	15	9	8	9	11

The point-out degree quantifies node radiation capability. Chengdu and Chongqing possesses relatively high point-out degrees, suggesting that their transportation, information flow and population migration exert a substantial influence on other cities, thereby enhancing the overall levels of urban traffic, information, and population migration within the CCUA. On the other hand, Zigong, Deyang, Leshan, Yibin, Meishan, Dazhou, Ya'an and Ziyang exhibit relatively low point-out degrees, indicating that these cities have limited impact on other cities in terms of traffic, information flow, and population migration. Due to their smaller economic size and weaker radiation capacities, their contributions to the overall improvement of urban transportation, information, and population migration levels within the CCUA are minimal.

#### 4.2.3 Betweenness centrality

In a network, each node has a unique betweenness centrality, with the node possessing the highest betweenness centrality occupying a core position. As indicated in Table 4, Chengdu consistently holds the betweenness centrality, underscoring its crucial role as a key connector among various cities within the CCUA. As one of the core cities, Chongqing ranks second in betweenness centrality, albeit with a considerable gap behind Chengdu. Therefore, it is vital to enhance the connections between Chongqing and other cities. However, cities other than Chengdu and Chongqing exhibit low betweenness centrality and sparse connections, emphasizing the need for stronger interconnections among them.

**Table 4:** The betweenness centrality of the CCUA.

City	Traffic flow	Information flow	Migration flow	Composite flow
Chengdu	130.833	121.833	154.667	142.833
Zigong	0	0	0.833	0
Luzhou	0	0	0.5	0.5
Deyang	0	0	0	0
Mianyang	1	1	0	1
Suining	0	0	0	0
Neijiang	0.333	0	1.833	1
Leshan	0.5	0	0	0
Nanchong	3	0	0	1.333
Meishan	0	0	0	0
Yibin	0	1.333	1.333	0
Guang'an	0	0	0	0
Dazhou	0	0	0	0
Ya'an	0	0	0	0
Ziyang	0	0	0	0
Chongqing	55.333	37.833	21.833	31.333

#### 4.2.4 Closeness centrality

Closeness centrality measures the extent to which a city operates independently of others. As illustrated in Table 5 concerning the composite flow, Chengdu takes the top spot in closeness centrality, indicating that it does not rely heavily on intermediate cities for communication. This allows Chengdu to achieve higher efficiency and enables other cities to seek direct communication with Chengdu, aiming to access more resources and foster further development. Chongqing and Nanchong secure the second and third positions, aligning with degree centrality rankings. The primary reason for this similarity is the presence of direct connections between these cities and others, reducing the reliance on intermediate cities for communication.

**Table 5:** The closeness centrality of the CCUA.

City	Traffic flow		Information flow		Migration flow		Composite flow	
	point-in degree	point-out degree	point-in degree	point-out degree	point-in degree	point-out degree	point-in degree	point-out degree
Chengdu	100	88.235	48.387	100	100	100	100	100
Zigong	44.118	50	36.585	55.556	55.556	55.556	55.556	53.571
Luzhou	42.857	57.692	37.5	51.724	55.556	55.556	55.556	55.556
Deyang	55.556	55.556	36.585	51.724	53.571	53.571	53.571	53.571
Mianyang	55.556	57.692	36.585	71.429	53.571	53.571	55.556	55.556
Suining	55.556	55.556	37.5	51.724	53.571	53.571	55.556	55.556
Neijiang	55.556	55.556	36.585	51.724	57.692	57.692	55.556	57.692
Leshan	55.556	50	35.714	51.724	53.571	53.571	53.571	53.571
Nanchong	60	57.692	36.585	62.5	55.556	55.556	57.692	60
Meishan	53.571	50	35.714	51.724	53.571	53.571	53.571	53.571
Yibin	55.556	48.387	37.5	60	55.556	57.692	55.556	53.571
Guang'an	53.571	55.556	37.5	51.724	55.556	55.556	55.556	55.556
Dazhou	55.556	50	37.5	53.571	53.571	53.571	55.556	53.571
Ya'an	51.724	48.387	53.571	6.25	51.724	51.724	51.724	51.724
Ziyang	55.556	53.571	35.714	51.724	55.556	55.556	55.556	53.571
Chongqing	71.429	83.333	40.541	100	71.429	68.182	71.429	78.947

### 4.3 Agglomerative subgroup analysis

**Fig. 5:**
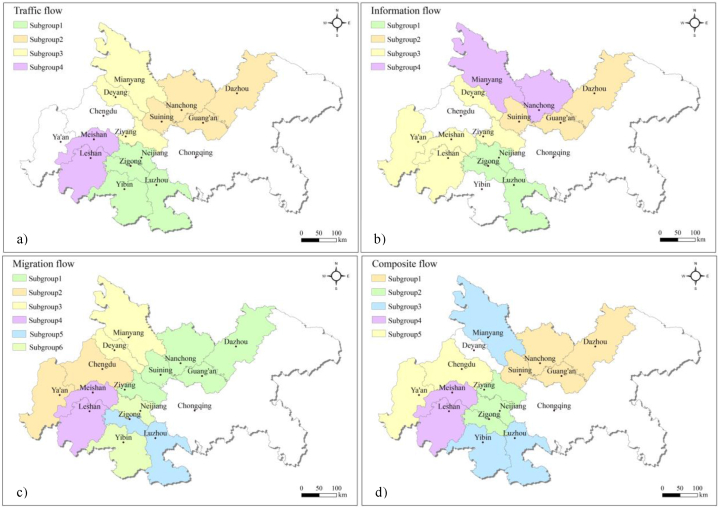
Analysis results for cohesive subgroups in the CCUA (a) The cohesive subgroups of traffic flow; b) The cohesive subgroups of information flow; c) The cohesive subgroups of migration flow; d) The cohesive subgroups of composite flow).

Cohesive subgroups represent cities that are closely interconnected within the city network, revealing clustering patterns within the network. Based on the analysis results shown in Fig. 5, the number of cohesive subgroups in the CCUA remains relatively stable, but their composition undergoes significantly changes. The traffic flow, information flow, migration flow, and composite flow networks consist of 4, 4, 6, and 5 cohesive subgroups, respectively.

In the traffic flow network, subgroup 1 (Luzhou, Neijiang, Yibin, Zigong), subgroup 2 (Guang'an, Nanchong, Suining, Dazhou), and subgroup 3 (Mianyang, Deyang, Ziyang) consist of 3 or 4 cities, highlighting close connections within each subgroup that contribute to the development of the CCUA. Subgroup 4 (Meishan, Leshan) comprises only two cities, exhibiting a simpler network structure and sparse connections. Chengdu and Chongqing, being the two core cities, enjoy better transportation accessibility and do not form cohesive subgroups with other cities. The connections between Ya'an and other cities are weak and require improvement.

In the information flow network, subgroup 1 (Zigong, Luzhou, Neijiang), subgroup 2 (Dazhou, Suining, Guang'an), and subgroup 3 (Ya'an, Deyang, Meishan, Ziyang, Leshan) consist of 3 or 4 cities, indicating strong connections within each subgroup. Subgroup 4 (Mianyang, Nanchong) comprises only 2 cities with a simpler network structure. Chengdu and Chongqing have closely connections with other cities and receive significant attention, not forming cohesive subgroups. Yibin receives relatively little attention and has limited connections with other cities, resulting in relative isolation within the subgroup.

In the migration flow network, subgroup 1 consists 5 cities, namely Nanchong, Guang'an, Dazhou, Suining, and Ziyang, indicating closely connections and high network density. Subgroup 2 (Chengdu, Ya'an), Subgroup 3 (Deyang, Mianyang), Subgroup 4 (Meishan, Leshan), subgroup 5 (Luzhou, Zigong), and subgroup 6 (Neijiang, Yibin) each contain 2 cities, suggesting relative sparse network connections. Chongqing, as a core city of the CCUA, has close connections with other cities and does not form a cohesive subgroup. Chengdu forms a cohesive subgroup with Ya'an, highlighting Chengdu's radiating and driving effect on Ya'an.

In the composite flow network, subgroup 1 comprises 4 cities (Guang'an, Nanchong, Suining and Dazhou). Subgroups 2 (Neijiang, Ziyang and Zigong) and 3 (Mianyang, Luzhou and Yibin) consist of 3 cities, showcasing close interconnections. Subgroups 4 (Meishan and Leshan) and 5 (Chengdu and Ya'an) contain two cities, with sparse connections between them. Chongqing and Deyang did not form cohesive subgroups.

Cities tend to form cohesive subgroups with nearby cities, and cities within a subgroup exhibit closer relationships than those outside the subgroup. Cohesive subgroups do not typically cross administrative boundaries, indicating limited cohesion between cities in Sichuan and Chongqing. In the CCUA, there are few core cities with strong spatial correlations, and isolated cities still exist. The subgroups are in the early stage of development with relatively simple structures. Therefore, it is crucial to strengthen cooperation between cities in the CCUA, leverage the leadership roles of Chongqing and Chengdu, and promote accelerated development in densely populated areas such as Zigong, Neijiang, Luzhou, Yibin, Nanchong, Suining, and Guang'an. Additionally, the cohesive subgroup structure within the CCUA should be optimized, enhancing connectivity, stability, narrowing the gaps between cities, and promoting balanced developmentd.

## 5 Conclusion and discussion

### 5.1 Conclusion

This study explores the urban network spatial structure in the CCUA from a multi-dimensional element flow perspective, yielding the following key findings:

(1) The composite flow network in the CCUA aligns closely with individual element flow network, particularly traffic flow. Each city exhibits varying connectivity levels with others at different element flow levels. The urban agglomeration demonstrates significant polarization, with Chengdu and Chongqing occupying dominant positions. Chengdu's influence surpasses that of Chongqing, reflecting a hierarchical distribution of “1 + 1+7 + 7". Connection strength varies spatial, with stronger connections in the west compared to the east. Besides Chengdu–Chongqing, other notable connections include Chengdu–deyang, Chengdu–Mianyang and Chengdu–Meishan, forming the primary level of connections. Overall, the urban spatial connections within the CCUA is relatively loose. Administrative boundaries constrain Chongqing's connections, limiting strong cross-regional ties.
(2) Element flow networks within the CCUA exhibit low density, indicating a loose overall structure and weak element connections. The flow space network of the CCUA is still in its early developmental stage. Chengdu demonstrates the highest connectivity, followed by Chongqing, while Ya'an exhibits the weakest connectivity. Chengdu and Chongqing exert significant influences and possess strong agglomeration capacities. Conversely, Deyang, Leshan, Meishan and Ya'an have limited connections and weaker agglomeration capacities. Chengdu and Chongqing have substantial impacts and strong radiation capacities, while Zigong, Deyang, Leshan, Yibin, Meishan, Dazhou, Ya'an and Ziyang have minimal influence and weak radiation capacities. For cities beyond Chengdu and Chongqing in the CCUA, centrality is at a lower-middle level, and the overall resource connectivity of the CCUA is weak.
(3) The CCUA consists of five cohesive subgroups: two subgroups with two cities, two subgroups with three cities, and one subgroup with four cities. The network density is low, and connections between cities are sparse. Cities tend to form cohesive subgroups with closer geographic proximity, leading to stronger connections within subgroups. Cohesion subgroups do not cross administrative boundaries, and Chongqing dose not form a cohesive subgroup with cities in Sichuan province. The CCUA lacks there core cities with high spatial correlations, and isolated cities persist. The subgroups are in the early stage of development, exhibiting a simple structural composition.

### 5.2 Discussion

Research on the spatial network structure of the CCUA has primarily focused on single-element flow analysis, emphasizing information flow, population migration flow and traffic flow. However, there is a lack of studies using a composite flow model to analyze the network connection patterns of the CCUA. Previous research based on information flow, using multi-year data to analyze changes in the spatial structure of the CCUA [22,23], has concluded that the connection between Chengdu and Chongqing is the strongest, while the strengths of connections between Chengdu and other cities are not consistent. In this study, we examine the three element flows and find that, in addition to Chengdu–Chongqing, Chengdu–deyang, Chengdu–Mianyang and Chengdu–Meishan also exhibit strong connections. The Cheng-De-Mian region, consisting Chengdu, Deyang, and Mianyang in Sichuan province, is characterized by advanced technology production, developed industries, high urbanization levels, and a significant contribution to GDP. Therefore, Chengdu has close connections with Deyang and Mianyang. Additionally, as part of the CCUA's development plan, the Chengdu metropolitan area, including Meishan, is being established. This initiative strengthens the connections between Chengdu and Meishan. Some studies suggest that Chengdu and Chongqing have similar level of connectivity when considering the overall element flow [27,40]. However, our findings reveal that Chengdu and Chongqing belong to different levels. Chengdu's radiation driving effect and influence are evidently greater than those of Chongqing, resulting in a city level distribution of “1 + 1+7 + 7”.

Due to constraints in administrative boundaries and the unavailability of district-level data for Chongqing, our study focuses solely on Chongqing as a research unit, neglecting connections between districts and counties within Chongqing and weakening the strength of connections in the eastern part of the Chengdu-Chongqing region. However, previous studies analyzing the spatial structure of the CCUA have yielded similar conclusions, indicating strong connections between Chengdu and the main urban area of Chongqing and weaker connections with other districts and counties in Chongqing [22,24,25,41]. Chongqing has not formed strong connections across administrative boundaries, as evidenced by weaker connections with nearby cities such as Dazhou and Luzhou. Although our study does not employ districts and counties as separate research units, our results to some extent reflect the spatial structure of the CCUA. The lack of district-level data for Chongqing may weaken connections within Chongqing itself, but its impact on the overall spatial pattern of the CCUA in our study is limited. We found that the eastern region exhibits stronger spatial connectivity compared to the western region. However, if district-level data for Chongqing were available, it would likely yield a similar connectivity pattern between the eastern and western regions. Besides Chengdu and Chongqing, cities within Sichuan province in the CCUA are closely connected, as are the districts within Chongqing. Our study concludes that Chongqing lacks strong cross-regional connections with other cities except for Chengdu. Consequently, districts within Chongqing would also lack strong connections with cities within Sichuan province in the CCUA. As the provincial capital, Chengdu maintains links with all the prefecture-level cities within the research area. While Chongqing is also a national-level central city with comparable comprehensive strength to Chengdu, administrative divisions limit its connections with prefecture-level cities within the research area, resulting in links primarily geographically close cities. Considering that the choice of spatial units affects the structure of urban networks and the sensitivity of urban networks to spatial units is a topic of urban network research, it is essential to conduct comparative studies analyzing urban network results at different spatial unit scales, such as prefecture-level units and county-level units.

Since Tencent's migration data are not public available, and complete data for the year 2019 is unattainable, we utilize 233 days of data. Most previous studies use average values over a specific time period to reflect city connections, while our study employs the sum of the 233-day data from 2019, providing a more representative approach to represent the spatial structure of the CCUA. Population migration network connections typically accompany traffic network connections since population movement requires transportation. Additionally, traffic network connections are often accompanied by information network connections, as individuals generally seek destination information before traveling. Therefore, information network connections have a certain supportive effect on transportation network connections. Future research should consider the overlapping nature of these three types of connection. Network connections between cities are influenced not only by information networks but also by traffic networks, population migration networks, and other factors. A comprehensive analysis of various networks can more accurately depict the spatial structure of an urban agglomeration.

Utilizing Baidu Index to represent the intensity of information flow between cities has limitations as it primarily reflect users’ online interest in cities. However, it still offers a visually intuitive representation of city network connections and aligns with the current trends of utilizing big data in research. On one hand, it is indeed worth noting that numerous studies have effectively utilized Baidu Index to analyze the spatial network structure characteristics of city clusters. These studies highlight the value and maturity of this approach in understanding urban connections [19,20]. On the other hand, in addition to information flow, we recognize the importance of incorporating two additional “flow spaces"–traffic flow and population migration flow. By integrating these elements, we aim to provide a comprehensive description of the connectivity network and status of cities within the CCUA. This inclusive approach serves to compensate for and rectify inherent biases in the information flow data itself, thereby ensuring a more comprehensive and accurate analysis of the urban connections.

The Chengdu Metropolitan Circle, centered around Chengdu and comprising Deyang, Meishan, and Ziyang, plays a crucial role in the development of the CCUA. Promoting the integration of Cheng-De-Mei-Zi is beneficial for the progress of the CCUA. From the perspective of composite flow connection strength, Chengdu-deyang and Chengdu-Meishan from the first level of connections, while Chengdu-Ziyang constitutes the second level, all exhibiting strong connections. In the future, it is essential to enhance Chengdu's role as a central city in terms of radiation and leadership, leverage the advantages of Deyang, Meishan, and Ziyang, and achieve coordinated development between large and small cities within the metropolitan circle. The spatial distribution of network connections between cities in the CCUA is unbalanced, yet a certain degree of spatial network structure has already formed. This study emphasizes that cities in the CCUA should not overlook the disparities among them while pursuing their individual development. For cities with weak element flow connections, it is crucial to more effectively embrace the radiation from core cities, consider their objective reality, make full use of their own advantages, and gradually narrow the gap with core cities. Chengdu and Chongqing still possess ample development space, and they should extend their radiation and influence to other cities enhancing their own growth and advancement.

## Data availability statement

Data will be made available on request.

## CRediT authorship contribution statement

**Xiaohui Zhang:** Writing – original draft, Visualization, Methodology, Formal analysis, Data curation. **Jifei Zhang:** Writing – review & editing, Writing – original draft, Visualization, Supervision, Resources, Project administration, Methodology, Funding acquisition, Formal analysis, Data curation, Conceptualization.

## Declaration of competing interest

The authors declare that they have no known competing financial interests or personal relationships that could have appeared to influence the work reported in this paper.
